# Lipid and Corticosteroid Biomarkers Under the Influence of Bisphosphonates

**DOI:** 10.1002/dta.3811

**Published:** 2024-10-15

**Authors:** Kathy Tou, Adam Cawley, Glenys Noble, Jaymie Loy, David Bishop, John Keledjian, Kireesan Sornalingam, Stacey Richards, Shanlin Fu

**Affiliations:** ^1^ Centre for Forensic Science University of Technology Sydney Sydney New South Wales Australia; ^2^ Racing Analytical Services Ltd Flemington Victoria Australia; ^3^ School of Veterinary Sciences Charles Sturt University Wagga Wagga New South Wales Australia; ^4^ Hyphenated Mass Spectrometry Laboratory University of Technology Sydney Sydney New South Wales Australia; ^5^ Australian Racing Forensic Laboratory Racing NSW Sydney New South Wales Australia

**Keywords:** anti‐doping, biomarkers, bisphosphonates, lipids, plasma

## Abstract

Detecting the use of bisphosphonates (BPs) in equine athletes is of interest to regulators and laboratories due to the threat to welfare issues for the potential to provide analgesic effects and manipulating bone structure. The detection of BPs in biological matrices is challenging due to erratic biological elimination and inconsistent analytical recoveries. Therefore, complementary approaches are needed to provide evidence of their misuse in racehorses. BPs have two sub‐classes: nitrogenous and non‐nitrogenous. This study investigated plasma elimination following administration of one example from each sub‐class, together with changes in endogenous eicosanoid and corticosteroids. Zoledronic acid (ZA) and tiludronic acid (TA) were administered by IV infusion to 8 thoroughbred horses with an 11‐month washout period between each administration. Sample preparation for quantification of BPs by liquid chromatography–tandem mass spectrometry (LC–MS/MS) utilised a two‐step solid phase extraction (SPE) consisting of polymeric reversed‐phase followed by weak anion exchange prior to derivatisation using trimethyl orthoacetate. Endogenous biomarkers were analysed after protein precipitation and SPE with polymeric reversed‐phase prior to liquid chromatography–high resolution mass spectrometry (LC‐HRMS) using data independent acquisition. The LC–MS/MS analysis showed ZA was undetectable after 8 h post‐administration while TA was detected up to the final collection point of 28 days post‐administration. The LC‐HRMS analysis utilised targeted (i.e., prior inclusion list of compounds) approaches to monitor level changes of eicosanoid and corticosteroid biomarkers. Putative biomarkers were identified and now subject to validation for translation into routine sample analysis for improved retrospectivity to detecting BP misuse in equine plasma.

## Graphical abstract

The detection of bisphosphonates is important due to the potential integrity and welfare issues associated with use of these substances. Targeted detection of a nitrogenous and non‐nitrogenous bisphosphonate was detected for 8 h and 28 days post‐administration, respectively. With this, the need for complementary indirect detection using lipid and corticosteroid biomarkers was investigated. Putative biomarkers were identified, which are now subject to validation into routine analysis for improved detection capabilities of bisphosphonate misuse in equine plasma.

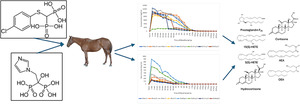

## 1 Introduction

Bisphosphonates (BPs) were first synthesised in the 1800s and are hydrophilic molecules that do not easily bypass the lipid membrane, are poorly absorbed through the gastrointestinal tract and strongly bind to hydroxyapatite molecules in areas of active bone resorption [1]. This has led to their use to treat bone disorders in humans (e.g., disorders involving the metabolism of calcium in humans) [2]. The use of BPs is of interest due to potential integrity and welfare issue associated with this class of drug in the horse [3, 4].

There are two classes of BPs, nitrogenous and non‐nitrogenous, with each having distinct modes of action [2, 5]. Nitrogenous BPs such as zoledronic acid (ZA) have the strongest affinity for hydroxyapatite with preferential localisation at sites of high bone turnover, are extremely potent and are commonly used in humans for the management of skeletal complications [6, 7]. The potency of nitrogenous BP is likely due to the carbon atom that contains the nitrogen‐containing side chain [6]. Comparatively non‐nitrogenous–based BPs such as tiludronic acid (TA), clodronate and etidronate are commonly used in humans for the treatment of Paget's disease [2]. In the equine system, non‐nitrogenous BPs inhibit the function of osteoclasts potentially interfering with adenosine triphosphate (ATP) in any one system and decreasing the amount of bone resorption in the horse [2, 8]. Compared to ZA, non‐nitrogenous BPs do not have the hydroxyl group on the carbon joining to the two phosphorus groups resulting in reduced potency and bone specificity [6]. Currently, only two non‐nitrogenous–based BPs (tiludronate and clodronate) are approved for the treatment of navicular syndrome, being a major cause of forelimb lameness in the horse [5]. According to the requirements set out by the International Agreement on Breeding, Racing and Wagering (IABRW) by the International Federation of Horseracing Authorities (IFHA) and AR 88A and AR 88‐AA in the Australian Rules of Racing, horses under the 4 years are prohibited from the administration of any BP both in‐ and out‐of‐competition so as to not interfere with the natural growth of the skeleton for younger horses [4, 5]. Regarding horses over the age of 4, horses subject to an administration of approved BPs are ineligible to compete until 30 clear days have passed since the administration. After this period, they are eligible to resume competition [9]. At present, nitrogen‐containing BPs are not approved for the use in racehorses; therefore, under the Rules of Racing, their presence in doping control samples would constitute a prohibited substance finding [5, 10].

BPs can persist in the bone for several years after administration as they have the capability to form hydroxyapatite crystals prior to being fully absorbed by active osteoclasts where their inhibitory action is performed [11]. Riggs et al. [4] in 2020 explored the concentration of TA in 24 horses following administration from approximately 1 month to over 3 years prior to date of sample collection. TA was detected in urine and plasma samples from all administered horses, including two that were administered TA more than 3 years prior. This could have serious ramifications for the racing industry if detected as there are many factors that would need to be considered prior to conviction. It is important to note that the concentrations of TA reported by Riggs et al. in horses with long post‐administration intervals were very low and their detections were erratic [4]. Therefore, the use of complementary testing to detect for biomarkers and the effects of TA would allow for a better understanding of how it may affect the horse.

The aims of the current study were to firstly, estimate the detection period for ZA and TA. Second, investigate lipid and/or corticosteroid biomarkers that could either complement or indirectly extend the detection time for a particular BP. Third, review biomarkers that display different responses to either BP studied in order to propose a differentiation strategy between a nitrogenous and a non‐nitrogenous BP administration.

## 2 Materials and Methods

### 2.1 Chemicals and Reagents

LC grade dichloromethane (DCM), hexane, hydrochloric acid (HCl), isopropanol (IPA), triethylamine (TEA) and trimethylorthoacetate (TMOA) were purchased from Merck (Castle Hill, NSW, Australia). Acetonitrile (ACN), formic acid (FA) and methanol (MeOH) of MS grade and ethanol (EtOH) of LC grade were purchased from ThermoFisher (Waltham, Massachusetts, USA). LC grade glacial acetic acid was purchased from ThermoFisher (Waltham, Massachusetts, USA) and water (H_2_O) used was ultrapure grade (18.2 MΩ.cm) obtained from a ThermoFisher Barnstead Smart2Pure system (Langenselbold, Hungary).

Certified reference material for the lipid biomarkers of 15(S)‐hydroxyeicosatetraenoic acid (15(S)‐HETE) (solution of 100 μg/mL), 18‐hydroxyeicosapentaenoic acid (18‐HEPE) (solution of 100 μg/mL), 5(S)‐hydroxyeicosatetraenoic acid (5(S)‐HETE) (solution of 100 μg/mL), arachidonoyl ethanolamide (AEA) (solution of 50,000 μg/mL), oleoyl ethanolamide (OEA) (5 mg powder), prostaglandin F_2α_ (PGF_2α_) (1 mg powder), 12(S)‐HETE‐D_8_ (solution of 100 μg/mL), 15‐HETE‐D_8_ (solution of 100 μg/mL), 5‐HETE‐D_8_ (solution of 100 μg/mL), oleoyl ethanolamide‐D_4_ (solution of 1000 μg/mL) and prostaglandin F_2α_‐D_4_ (solution of 100 μg/mL) manufactured by Cayman Chemicals (Ann Arbor, Michigan, USA) were purchased from Sapphire BioScience (Redfern, NSW, Australia). Corticosteroid biomarkers hydrocortisone (HC) (solution of 950 μg/mL) and cortisone (C) (solution of 1.034 mg/mL) were obtained from Merck (Castle Hill, NSW, Australia) and the internal standard hydrocortisone‐D_4_ (solution of 1.009 mg/mL) was from Cambridge Isotope Laboratories (Andover, Massachusetts, USA). 18‐Hydroxycortisol (1 mg powder) manufactured by IsoSciences (Ambler, Pennsylvania, USA) was purchased from PM Separations (Capalaba, QLD, Australia).

Two sources of TA were obtained for either calibration or quality control. For calibrators, TA was manufactured and purchased from Merck (Castle Hill, NSW, Australia). For quality control spikes and the corresponding internal standard, TA, TA‐D_5_, ZA and the corresponding internal standard, ZA‐^13^C_2_^15^N_2_ were manufactured by Toronto Research Chemicals (Toronto, Ontario, Canada) and purchased from PM Separations (Capalaba, QLD, Australia).

### 2.2 Administration Study—TA and ZA

An eight‐horse study was completed using an intravenous (IV) infusion of ZA through the jugular vein in the neck of the horse for 30 min. A dose of 0.057 mg/kg of ZA (Permit number: 81385. Randlab, Chipping North, NSW, Australia) was administered through one jugular catheter with blood samples taken via the opposite jugular vein to avoid cross contamination. On a separate occasion 11 months later, a dose of 1.0 mg/kg of TA (*Tildren*, Ceva Animal Health. Glenorie, NSW, Australia) was injected by a 30‐min IV infusion into the same location as the ZA administration using the same 8 horses. For a pre‐administration samples, samples were taken at time of administration for each horse (Time 0). Subsequent blood samples were then collected at 1, 5, 10, 15, 20, 30, 45, 60, and 90 min, 2, 3, 4, 6, 8, and 12 h, and 1,2, 3, 4, 5, 6, 7, 14, 21‐ and 28‐days post‐infusion. It is important to note that blood samples were taken from 1 min after the entire amount of drug had been given to each horse. Blood samples were then immediately centrifuged to obtain the plasma and stored at −20°C until analysis. Animal ethics approval (ZA: A20062, TA: A21362) was obtained for these administrations from Charles Sturt University Animal Care and Ethics Committee and reviewed by the Racing NSW Animal Care and Ethics Committee.

### 2.3 BP Extraction

The method was adapted from the previous work of Popot et al. [8] and Wong et al [10] and is the National Association of Testing Authorities (NATA) accredited qualitative method at the Australian Racing Forensic Laboratory (ARFL) in accordance with the ISO/IEC 17025 standard. An aliquot of 1 mL of equine plasma had relevant internal standard (IS) added at a concentration of 20 ng/mL for TA‐D_5_ and 50 ng/mL for ZA‐^13^C_2_^15^N_2_. Samples were pH adjusted to 4 using 2 mL of buffer containing diluted HCl in H_2_O (pH of 2) then centrifuged at 3000 rpm for 10 min.

Solid phase extraction (SPE) was completed using the Biotage Extrahera Classic (Uppsala, Sweden) with two separate cartridges. The first SPE was performed with a Waters Oasis HLB (60 mg 3 mL) cartridge (Milford, Massachusetts, USA) for TA or an Agilent Bond Elut Polypropylene (PPL, 100 mg, 3 mL) cartridge (Santa Clara, California, USA) for ZA. The cartridge was conditioned using MeOH (2 mL) and H_2_O (2 mL) prior to samples being loaded and collection of the flow‐through for the second SPE. The second SPE for both TA and ZA used a Waters Oasis® WAX (60 mg, 3 mL) cartridge (Milford, Massachusetts, USA). The cartridge was conditioned with MeOH (2 mL) and acidified water (pH adjusted to 4 with FA; 2 mL). Samples were loaded then washed with acidified water (pH adjusted to 4 with FA; 2 mL) followed by MeOH (2 mL). Cartridges were dried for 2 min before target compounds were eluted with 15% TEA in MeOH (3 mL). The eluent was dried under nitrogen gas at 60°C.

Samples were derivatised using acetic acid and TMOA with heating at 95°C for 60 min before being dried under nitrogen gas at 60°C. Samples were reconstituted in 50:50 MeOH and H_2_O (100 μL) and stored at 4°C until liquid chromatography tandem mass spectrometry (LC–MS/MS) analysis.

### 2.4 Instrument Parameters for BPs

Using the NATA accredited qualitative method for BP analyses, LC–MS/MS analysis was undertaken with a LC30 liquid chromatograph coupled to an 8050‐mass spectrometer from Shimadzu Scientific Instruments (Kyoto, Japan). Separation was performed using a Waters XBridge C18 column (2.1 mm × 150 mm, 3.5 μm) (Milford, Massachusetts, USA) using a gradient elution. Aqueous mobile phase A consisted of 0.1% FA in H_2_O whilst organic mobile phase B was 0.1% FA in ACN with a run time of 14 min. The gradient was 0–1 min B (2%), 1–8 min B (98%), 8–12 min B (2%) and then kept constant till 14 min. The flow rate was constant at 0.2 mL/min with an injection volume of 5 μL, and the column oven was set to 35°C.

Shimadzu LabSolutions software (version: 5.93) was used for data acquisition with multiple reaction monitoring (MRM) in ESI positive mode (Table 1).

**TABLE 1:** Optimised mass spectrometric conditions for bisphosphonates.

Compound	Precursor ion (*m/z*)	Product ion (*m/z*)	Collision energy (CE) (V)
Tiludronic acid	375.00	342.85	17
		157.00	21
		216.95^a^	21
		154.80	44
Tiludronic acid‐D_5_	378.90	161.00^a^	22
		86.95	22
Zoledronic acid	328.80	202.90^a^	17
		135.00	13
		171.10	17
Zoledronic acid‐^13^C_2_^15^N_2_	333.10	206.90^a^	21
		136.95	30

^a^ Product ion used to quantify.

### 2.5 Method Validation Parameters for TA and ZA

The established LC–MS/MS method for the detection of TA and ZA was validated for quantification from 1 mL of equine plasma. The parameters of linearity, sensitivity, accuracy, precision, recovery, matrix effects, dilution and stability were assessed. Limit of detection for sensitivity was assessed between 0.05 and 0.5 ng/mL for TA whilst ZA was assessed between 5 and 20 ng/mL. Linearity was assessed with a low concentration (1.0–200 ng/mL) and high concentration (200–1000 ng/mL) range for TA and between 20 and 1000 ng/mL for ZA. For accuracy, precision, recovery, matrix effects and stability, TA was assessed at 10 ng/mL whilst ZA was assessed at 50 ng/mL. To assess the effects of diluting the plasma, a 200 ng/mL TA spiked plasma sample was diluted 1:10 in buffer and a 100 ng/mL ZA spiked plasma sample was diluted 1:2 in buffer containing diluted HCl in H_2_O (pH of 2). Stability was assessed at two storage conditions, 4°C and −20°C spiked in equine plasma over a 4‐week period.

### 2.6 Data Analysis Parameters for BPs

Data was processed using Shimadzu Insight software (version 3.2) with further analysis using Excel (version: 16.71). This method includes each compound, the precursor and product ion for MRM transition and the IS used for compound quantification. The concentrations were calculated using linear regression with the calibration curve being the area ratio of the target compound to the internal standard response i.e. TA/TA‐D_5_ and ZA/ZA‐^13^C_2_^15^N_2_ for the respective concentration.

### 2.7 Surrogate Matrix for Biomarkers

A surrogate matrix was utilised due to the endogenous nature of the target compounds. This surrogate matrix was made using plasma pooled from a collection of equine plasma having gone through the routine analysis at the ARFL and determined not to contain any exogenous drugs. Liquid–liquid extraction was performed using 3‐mL plasma aliquots and DCM/EtOH (90:10 v/v, 4 mL) in a DWK Life Sciences screw top kimble tube (Wertheim, Germany). Solutions were rotated for 20 min at medium speed allowing for mixing between layers. Each tube was centrifuged at 3000 rpm for 10 min before the aqueous plasma layer was transferred into glass tubes and stored at −20°C until use.

### 2.8 Biomarker Extraction

The method was adapted from Toewe et al. for analysis of lipid mediators in human plasma [12]. An aliquot (100 μL) of plasma was transferred into a 1.5 mL microcentrifuge tube for protein precipitation with 300 μL of 0.1% FA in MeOH. A mixed working solution of the various lipid internal standards (IS) were made to concentrations of 60 ng/mL for PGF_2α_‐D_4_, 15‐HETE‐D_8_, 5‐HETE‐D_8_, 12‐HETE‐D_8_, and 12 ng/mL for OEA‐D_4_ with 10‐μL spiked into each sample. The corticosteroid IS HC‐D_4_ was made separately to 50 ng/mL with 10 μL also added to each sample. Samples were then subject to mixing for 3 min at 4°C and centrifugation at 13,000 rpm for 10 min. To each supernatant, 900 μL of 0.1% FA in in H_2_O was added to the supernatant and further agitated before SPE.

SPE was completed on a UCT positive pressure manifold using Phenomenex Strata‐X 10 mg reversed phase SPE cartridges (Torrance, California, USA). The cartridge was conditioned with 0.1% FA in MeOH (1 mL) and 0.1% FA in H_2_O (1 mL). Sample was loaded before washing with 0.1% FA in H_2_O (1 mL), 0.1% FA in 15% EtOH (1 mL) and hexane (1 mL). The cartridge was then eluted with 300 μL of 0.1% FA in MeOH. Each sample was dried using the Genevac EZ‐2 evaporator set to 45°C for 90 min. Samples were reconstituted in 100 μL in 0.1% FA in MeOH in an autosampler vial and stored at −20°C until LC‐HRMS analysis.

### 2.9 Instrument Parameters for Biomarkers

LC‐HRMS analysis was performed using an LC40 system coupled to a 9030 quadrupole‐time of flight (QTOF) mass spectrometer from Shimadzu Scientific Instruments (Kyoto, Japan). Separation used a Phenomenex Kinetex C8 column (2.1 mm × 150 mm, 2.6 μm) (Torrance, California, USA) with gradient elution. Aqueous mobile phase A consisted of 0.1% FA in H_2_O, and organic mobile phase B is ACN. The gradient was 0 min B (10%), 0–5 min B (25%), 5–10 min B (35%), 10–20 min B (75%), 20–28 min B (98%) and 28–30 min (10%) as post equilibration time. The flow rate was constant at 0.4 mL/min with an injection volume of 5 μL and column oven set to 40°C. The column, mobile phase and gradient were previously developed and optimised for the detection of lipid mediators [12, 13].

Shimadzu LabSolutions software (Version: 5.99 SP2) was used for data acquisition. MS data was collected between 1 to 25 min using data independent acquisition (DIA) sequentially in ESI+ and ESI− modes with the *m/z* range between 50 and 700.

### 2.10 Data Analysis Parameters for Biomarkers

Data was processed using Shimadzu Insight Explore software (Version: 3.8 SP1) and Excel (version 16.71) with review of the precursor ion, relevant product ions and the relative internal standard. The concentrations were calculated using linear regression with the calibration curve being the area ratio of the target compound to the corresponding internal standard response.

### 2.11 Method Validation Parameters for Biomarkers

Biomarker analysis was validated for 100 μL of equine plasma using the surrogate matrix as described in Section 2.6—surrogate matrix for biomarkers. This was due to the endogenous nature of biomarkers including oleoyl ethanolamide (OEA), arachidonoyl ethanolamide (AEA), hydrocortisone (HC) and cortisone (C). Concentrations for method validation were set at 50 ng/mL for HC, 5 ng/mL for C and OEA and 1 ng/mL for AEA for precision, accuracy, recovery and matrix effects with linearity ranges from 0.2 to 50 ng/mL for OEA, AEA and C and 0.2 to 200 ng/mL for HC.

## 3 Result and Discussion

### 3.1 Method Validation of TA and ZA

Following linearity assessments, *R* ^2^ values were greater than 0.99 for the low (1.0–200 ng/mL) and high (200–1000 ng/mL) range calibrations for TA and for ZA (20–1000 ng/mL). Samples with analytes exceeding 1000 ng/mL were repeated at a lower sample volume to ensure analytes were within the calibration range. The LOD and LOQ was estimated to be 0.5 and 1.0 ng/mL for TA and 10 and 20 ng/mL for ZA, respectively.

For accuracy and precision, the percentage relative error and percentage relative standard deviation were within 20%. However, recovery was poor for TA (13.2%) and ZA (2.70%), which is a likely contributing factor to the high LOD and LOQ for ZA. A low recovery of 22.5% for ZA was also reported by Wong et al. [10]. This is assumed to be the use of an inefficient cartridge for the second SPE. According to unpublished results by Klingberg et al. (short communication) an improved cartridge for BP extraction is the Affinisep AttractSPE WAX (Normandy, France). The exact reason for such a low recovery remains unknown; however, it may be likely due to the low pH which the sample are isolated on the cartridge. A higher pH (e.g., 4 to 6) could be investigated for improved recoveries. Regarding matrix effects, both compounds showed only slight ion suppression.

Dilution was assessed for TA and ZA with the percentage relative error at 23% with a 1 in 10 dilution and 6% with a 1 in 2 dilution deeming both acceptable. TA and ZA were stable at both 4°C and −20°C throughout the 4‐week period; however, TA did display minor degradation at the 4‐week time point.

### 3.2 Biomarkers to Be Monitored

The lipid biomarkers of inflammation from the arachidonic acid (AA) cascade (e.g., oleoyl ethanolamide [OEA] and arachidonoyl ethanolamide [AEA]) and stress‐related corticosteroids (such as hydrocortisone/cortisol and cortisone) were chosen for monitoring in this study. Lipid biomarkers have historically been shown to have an effect under the administration of exogenous corticosteroids, non‐steroidal anti‐inflammatories and cannabidiol as summarised by Tou et al. [14]. Following on from this, certain lipids in the AA cascade have been known to show anti‐inflammatory properties under the influence of non‐nitrogenous BPs [15, 16, 17].This poses the question of whether other lipid biomarkers in the same cascade could potentially be used for the detection of BPs. Stress‐related corticosteroids have always been of interest in the racing industry as it poses an integrity issue of whether horses should be racing if they are experiencing pain. Stress and inflammatory biomarkers such as HC and C are expected to increase when the horse has been injured [18, 19, 20]. Whilst an increase in response is expected in an injured horse, the decrease in response of these two biomarkers may also indicate an administration of a substance to minimise pain and/or inflammation in the horse.

The use of biomarker ratios has also been applied to antidoping in both human and equine sports [21]. In equine sports, biomarker ratios have been utilised to detect steroid misuse in addition to when thresholds have been absent. As reported previously [22], HC is controlled in urine with the urinary threshold of 1 μg/mL, but there is no threshold for equine plasma. The use of the biomarker ratio between HC and C in equine plasma was developed as a non‐targeted screening method for corticosteroid administrations [22]. This ratio was utilised as C was considered a stable biomarker not exceeding 13 ng/mL deeming this biomarker appropriate as an endogenous reference compound (ERC) [21]. The ratio between HC and C has been previously investigated [22] due to HC being susceptible to circadian variations. Therefore, the ratio between these two markers could potentially normalise an individual horse's variability. The potential for use of biomarker ratios was also further explored in this study using OEA and AEA. These two biomarkers are analogues of each other and derivatives of the main precursor of AA, therefore the OEA/AEA ratio was monitored throughout the BP administrations.

Biomarkers not quantitatively validated provided peak area responses instead of the calculated concentration. Following linearity assessments for all four biomarkers (0.2–50 ng/mL for OEA, AEA and C and 0.2–200 ng/mL for HC), *R* ^2^ values were all greater than 0.99 with y‐residual plots showing no evidence of bias amongst the spike samples. LOD and LOQ were estimated for the four monitored biomarkers (Table 2). This is difficult for OEA and HC due to endogenous content present in the surrogate matrix. Results for accuracy, precision, recovery and matrix effects are provided in Table 3. The possibility of endogenous interference in addition to inconsistent spiking at the lower concentrations could account for the higher relative error affecting the accuracy of AEA and HC determinations. Due to the extremely high percentage relative error for AEA, peak area was utilised for further analysis. In addition, the results for matrix effects indicate all compounds were affected by ion suppression.

**TABLE 2:** LOD and LOQ for biomarker method validation.

	LOD (ng/mL)	LOQ (ng/mL)
OEA	< 0.1	0.2
AEA	0.1	0.2
HC	< 0.1	0.2
C	0.1	0.2

**TABLE 3:** Accuracy, precision, recovery and matrix effects for biomarker method validation.

	Concentration (ng/mL)	Accuracy (% RE)	Precision (% RSD)	Recovery (%)	Matrix effects (%)
OEA	5.00	2.00	6.80	82.0	71.0
AEA	1.00	68.4	18.0	79.0	93.0
HC	50.0	33.0	6.00	76.0	72.0
C	5.00	13.0	11.0	91.0	62.0

Stability was assessed over a 4‐week period in duplicate with samples being extracted and analysed at the 2‐week, 3‐week and 4‐week mark. All 4 compounds were stable over the 4‐week period.

### 3.3 ZA Administration Study

**FIGURE 1:**
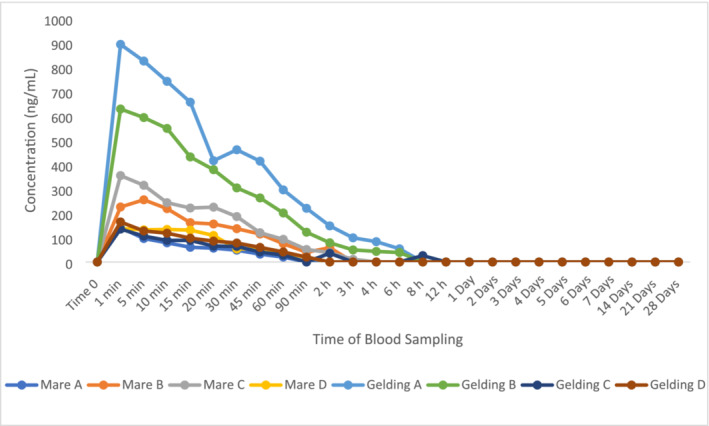
Plasma elimination of ZA administered to eight horses.

The plasma elimination profiles of ZA in eight horses (four mares and four geldings) are shown in Figure 1.

All eight horses exhibited a reduction of plasma ZA following the maximum concentration usually at 1 or 5 min post administration. The highest concentration was 894 ng/mL in Gelding A with the lowest maximum concentration being 135 ng/mL in Gelding C. The longest detection period for ZA was observed to be 8 h post‐administration, which is consistent with the results as published by Nieto et al. [6].

This relatively short window of detection highlights the need to search for biomarkers capable of indirectly extending the time of detection for the administration of a nitrogenous BP. Under the influence of ZA, the following biomarkers displayed notable change: OEA, AEA/OEA ratio, HC and HC/C. The biomarkers that remained stable following ZA administration were AEA, cortisone, 18‐hydroxycortisol and 18‐HEPE (Figures S1–S4). All other monitored biomarkers were not detected throughout the administration period.

**FIGURE 2:**
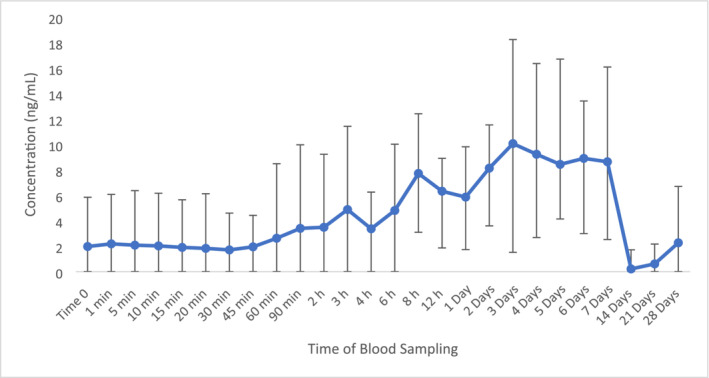
Average concentration of plasma OEA following ZA administration (*n* = 8) with vertical bars representing the range.

Concentrations of OEA were quantified throughout the administration period and remained stable for up to 60 min post‐administration as seen in Figure 2. OEA displayed up‐regulation by 337% at 7 days post‐administration compared to time 0. However, this was followed by 90% decrease at 14 days post‐administration compared to time 0 and a return to basal levels. Notwithstanding this, these time points were not deemed significant in comparison to time 0 as the *p*‐values were > 0.05.

**FIGURE 3:**
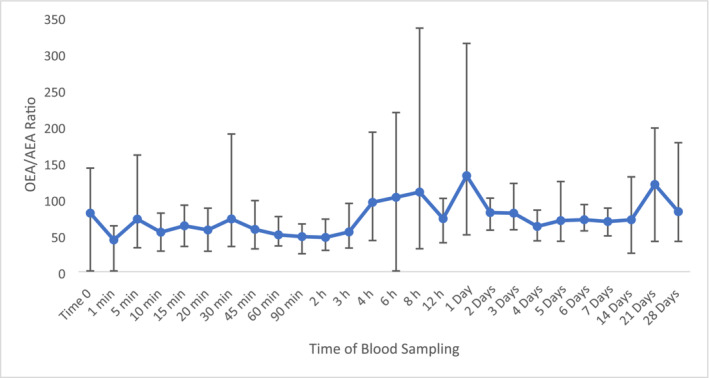
Average plasma OEA/AEA values following ZA administration (*n* = 8) with vertical bars representing the range.

Due to the high percentage relative error with AEA, the peak area was used for the analysis of the OEA/AEA ratio. The displayed change at 21 days post‐administration in comparison to time 0 with an increase to a value of 119 (Figure 3). An apparent bimodal pattern was observed at 1 day post administration with an increase of 63% with subsequent increase (48%) observed at 21 days. However, these changes were not considered significantly different to time 0 as the calculated fold change was 0.02.

**FIGURE 4:**
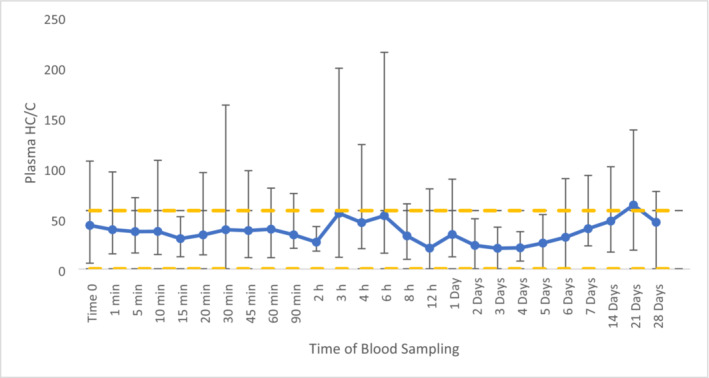
Average plasma HC/C values following ZA administration (*n* = 8) (yellow dotted line indicates PRL of 58) with vertical bars representing the range.

Cortisone displayed stable concentrations not exceeding 2.5 ng/mL (Figure S2), providing the opportunity for it to be used as an endogenous reference compound (ERC). HC/C values were relatively stable throughout the administration period with the exception between 2 and 8 h post‐administration (Figure 4). At 21 days post‐administration, there was an increase in HC/C values but with a fold‐change of only 1.0. From previous research investigating HC/C values in equine plasma, an upper and lower population reference limit of 58 and 0.24 were proposed [22]. Utilising these, at 21 days post‐administration, the HC/C value of 64 also exceeds the proposed upper ratio threshold of 58 [22], highlighting this sample would be flagged as abnormal from routine screening. With HC/C providing time points surpassing the population reference limit, there is the possibility of using HC/C as a biomarker that can distinguish between TA and ZA administrations.

### 3.4 TA Administration Study

**FIGURE 5:**
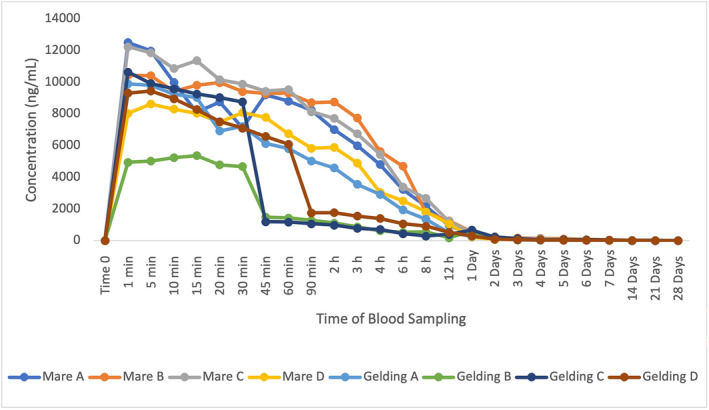
Plasma elimination of TA in 8 administered horses.

TA was administered to eight horses, (four being mares and four geldings). Plasma concentrations of TA in these horses are shown in Figure 5.

**FIGURE 6:**
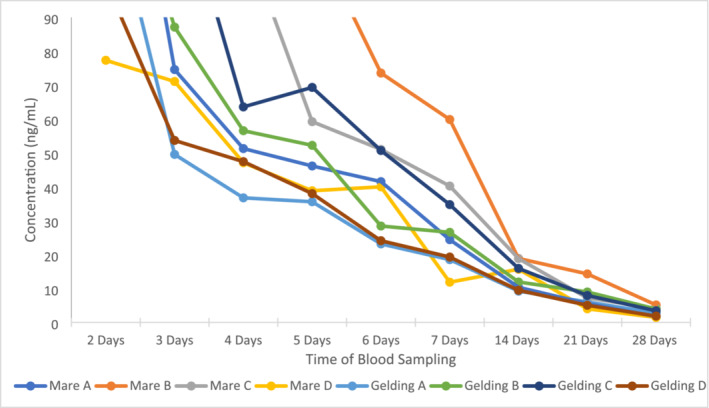
Plasma elimination of TA in 8 administered horses (2–28 days post‐administration).

All eight horses displayed a reduction in plasma TA after the maximum concentration at 1 min post‐administration. The highest concentration was estimated to be 12,500 ng/mL from Mare A, and the lowest maximum concentration was 5373 ng/mL Sfrom Gelding B, illustrating large inter‐individual variation of TA. Figure 5 shows a rapid decline in the TA concentration over the first 2 days post‐administration. Figure 6 removes the high concentrations obtained after initial administration to show that TA is detected and quantified at the LOQ of 1 ng/mL at 28 days post‐administration.

These results are consistent with those presented by Popot et al. [23], where TA was detected 15 days post‐administration at less than 10 ng/mL. Similarly, another study by Popot et al. [8] showed plasma concentrations down to 2.5 ng/mL up to 30 days post‐administration. There is the possibility of TA being detectable for longer periods of time as reported by Riggs et al. [4]. The limitation of our study presented here is the lack of sampling post 28 days.

With the longer detection period but low concentrations, complementary analysis of lipid and corticosteroid biomarkers was investigated. The following compounds demonstrated measurable change: Prostaglandin F_2α_, 15(S)‐HETE/5(S)‐HETE ratio, OEA/AEA ratio, OEA and 18‐HEPE. Biomarkers that remained static include AEA and HC/C (Figures S6 and S7, respectively).

**FIGURE 7:**
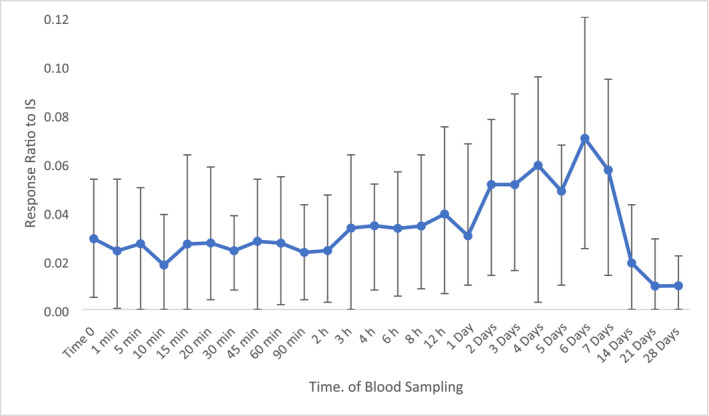
Average peak area response ratio for plasma PGF_2α_ following TA administration (*n* = 8) with vertical bars representing the range.

Prostaglandin F_2α_ (PGF_2α_) demonstrated the most interesting plasma profile following TA administration. Figure 7 shows an increase from 2 to 7 days post‐administration. Fold changes of greater than 1.5 with *p*‐values of less than 0.05 were estimated for these time points with comparison to time 0. Rapid decrease was then observed between 14 days and 21 days post‐administration.

The biological relevance of PGF_2α_ is hypothesised to be relevant to the anti‐inflammatory activity that a non‐nitrogenous BP displays in humans. This is caused by the inhibition of the release of inflammatory mediators from activated macrophages (white blood cells at the site of infection) [15]. These inflammatory mediators include Interleukin‐1 (IL‐1) [16]. In humans evidence of IL‐1 stimulated chondrocytes (cells responsible for cartilage formation) [17] may be related to the synthesis of PGF_2α_ which could explain the sharp increase observed at 6 days post‐administration for this biomarker.

**FIGURE 8:**
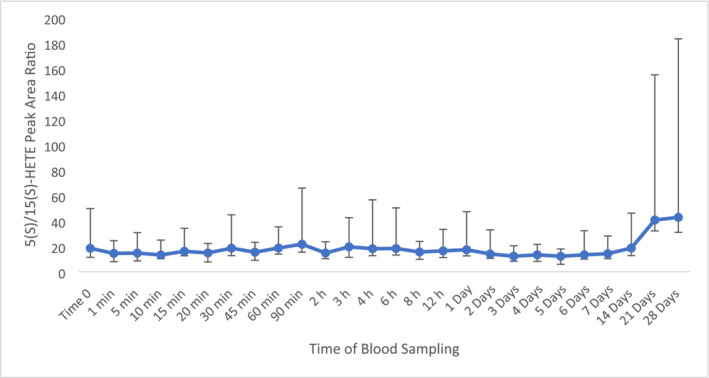
Average peak area ratio for plasma 5(S)‐HETE to 15(S)‐HETE following TA administration (*n* = 8) with vertical bars representing the range.

The 5(S)‐HETE to 15(S)‐HETE ratio was investigated as a normalised biomarker. The integrated peak area of the two biomarkers was used since both were not quantifiable. The ratio appeared to be relatively stable up to 7 days post‐administration with a percentage change not higher than 35%. Between 14 and 28 days post‐administration, significant increase was observed with percentage changes of 120 and 132%, fold changes of 1.5 and 1.6 with associated *p*‐values less than 0.05 (Figure 8).

Little is known about the biological relevance of both 5(S)‐HETE and 15(S)‐HETE in the equine system nor in humans under the influence of BPs. However, in relation to the AA cascade, 15(S)‐HETE is derived from the 15‐LOX enzyme. With a decrease of 15(S)‐HETE, it can be hypothesised that the 15‐LOX enzyme is inhibited to metabolise AA into 15(S)‐HETE [24, 25, 26].

**FIGURE 9:**
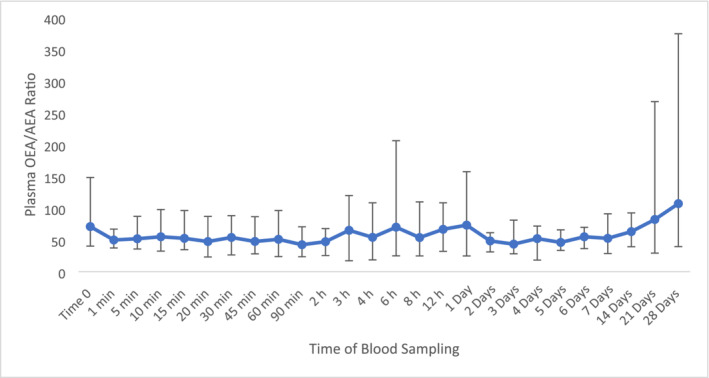
Average plasma OEA/AEA values following TA administration (*n* = 8) with vertical bars representing the range.

OEA was also quantifiable throughout the administration period with concentrations observed up to 4 ng/mL. There was no consistent pattern for OEA (Figure S5) from time 0; however, at 28 days post‐administration, there was a concentration decrease of 33% but no significant difference in comparison to pre‐administration samples. The ratio of AEA and OEA was investigated using the peak area due to the high percentage relative error with AEA. Four geldings showed a consistent pattern; however the mares were less consistent due to some unquantifiable concentrations for AEA, so the peak area was used. Figure 9 shows an increase of 16% at 21 days post‐administration and 50% at 28 days post‐administration; however, this was not significantly different compared to time 0. Nevertheless, following the increase observed at later time points, this ratio could potentially be useful to monitor for identifying prior TA administrations.

The concentration of 18‐HEPE was decreased at 14 days post‐administration by 36% in comparison to time 0. This pattern continued during 21 to 28 days post‐administration with concentrations being reduced by 26% and 55%, respectively (Figure S8).

### 3.5 Comparison Between the TA and ZA Administration Studies

**FIGURE 10:**
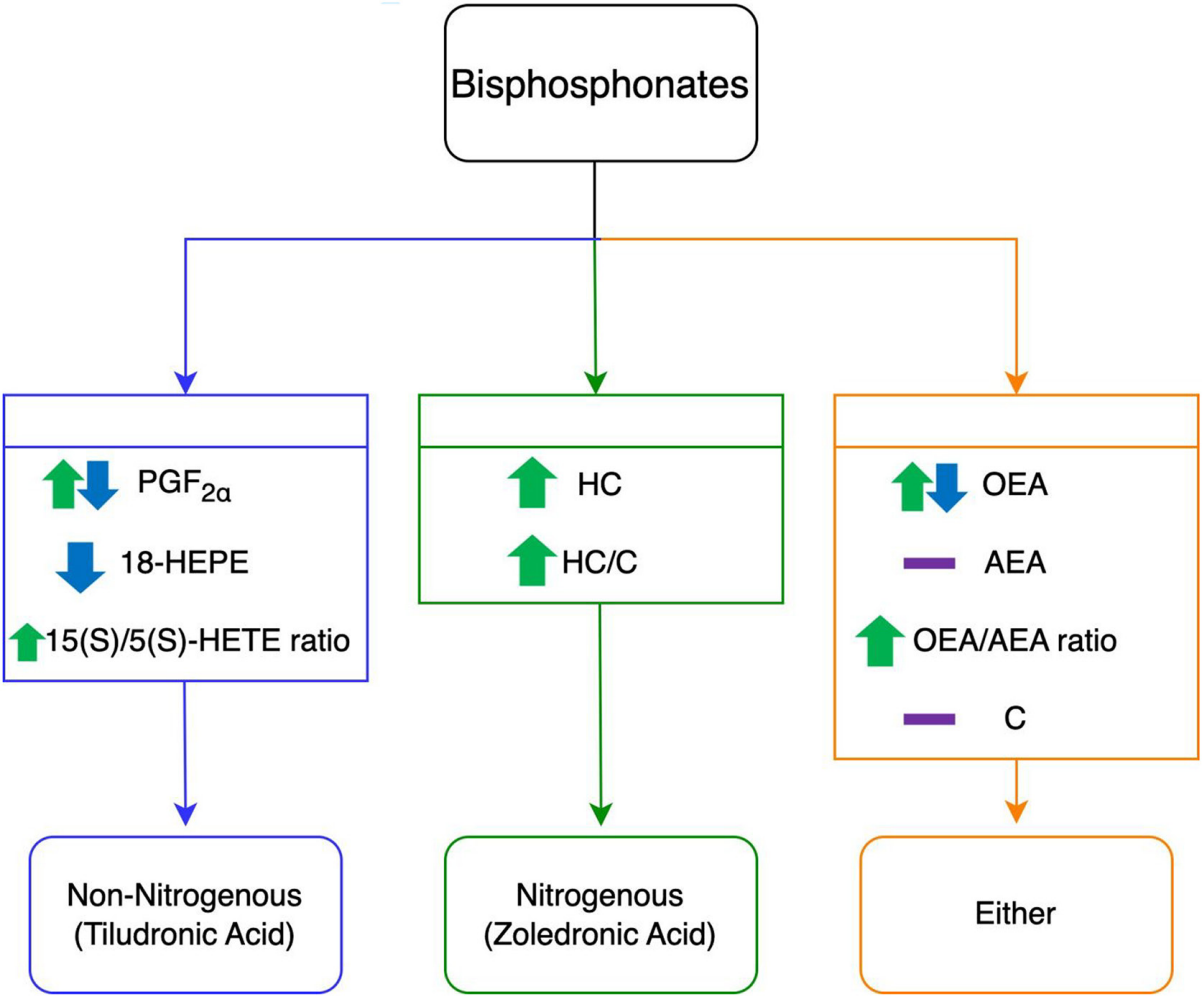
Proposed decision strategy using lipid and corticosteroid biomarkers for differentiation of TA and ZA administrations.

The biomarker results from TA and ZA administrations were reviewed for differentiation between a nitrogenous and a non‐nitrogenous BP administration (Figure 10). With each administration, different compounds either complemented or indirectly extended the detection period. For TA, whilst the parent drug itself was detectable up to 28 days post‐administration, it must be noted that as indicated in literature by Riggs et al., the detection of TA over extended periods of time can be deemed erratic. Therefore, the detection of PGF_2α_ (increase and rapid decrease), 18‐HEPE (decrease) and the 15(S)/5(S)‐HETE (increase) provided evidence of effect up to 28 days post‐administration. Comparatively, ZA was only detectable up to 8 h post‐administration. Therefore, indirect detection with biomarkers can be advantageous to extend the time of detection up to 21 days post‐administration. OEA was increased and then further decreased in plasma levels extending the detection time to 14 days post‐administration. Used in combination, OEA/AEA (decrease), HC (increase) and HC/C (increase) extended the detection time up to 21 days post‐administration. These biomarkers being specific to each administration with either complementary or indirect detection, would be beneficial for analysts to determine which type of BP was administered.

### 3.6 Study Limitations

The use of biomarkers is currently a novel technique in the equine racing industry given that routine analysis relies on the detection of the prohibited substance [27]. Therefore, this approach would only be a complementary technique to the traditional detection of the prohibited substance and not as a full replacement. This study is considered preliminary for the indirect detection of BPs. The validity of the proposed potential biomarkers needs to be further investigated by additional administration studies in different jurisdictions to explore alternative biomarkers to complement existing detection measures.

## 4 Conclusions

Using a targeted LC–MS/MS method, ZA and TA were detectable up to 8 h and 28 days post‐administration respectively, consistent with the literature. To extend the surveillance capability for BPs, lipid and corticosteroid biomarkers provided a complementary indirect method using LC‐HRMS. Specifically, PGF_2α_, 18‐HEPE and 15(S)/5(S)‐HETE values have the potential to provide evidence of TA administration, while HC and HC/C values show potential to provide evidence of ZA administration. Biomarkers altered following BP administration were OEA, AEA, OEA/AEA and C. The decision strategy presented here can be further expanded to investigate the effects of other BPs on lipid and corticosteroid biomarkers.

## Conflicts of Interest

The Shimadzu LC‐QTOF 9030 was a demonstration instrument provided by Shimadzu Scientific Instruments (Australasia) to the ARFL for evaluation of biomarker profiling in equine anti‐doping.

## Supporting information


**Figure S1.** Average integrated peak area in plasma for AEA following ZA administration (*n* = 8) with vertical bars representing the range.
**Figure S2.** Average concentration of plasma cortisone following ZA administration (*n* = 8) with vertical bars representing the range.
**Figure S3.** Average integrated peak area in plasma for 18‐hydroxycortisol following ZA administration (*n* = 8) with vertical bars representing the range.
**Figure S4.** Average concentration of plasma 18‐HEPE following ZA administration (*n* = 8) with vertical bars representing the range.
**Figure S5.** Average concentration of plasma OEA following TA administration (*n* = 8) with vertical bars representing the range.
**Figure S6.** Average integrated peak area in plasma for AEA following TA administration (*n* = 8) with vertical bars representing the range.
**Figure S7.** Average concentration ratio for plasma HC/C following TA administration (*n* = 8) with vertical bars representing the range.
**Figure S8.** Average concentration of plasma 18‐HEPE following TA administration (*n* = 8) with vertical bars representing the range.
