# Supplementary material for: Lipid and Corticosteroid Biomarkers Under the Influence of Bisphosphonates
Source: Drug Test Anal. 2024 Oct 15;17(7):1107–17. doi: 10.1002/dta.3811 (PMC12209688; doi:10.1002/dta.3811)
Supplement: Supplementary file 1 — Figure S1. Average integrated peak area in plasma for AEA following ZA administration (n = 8) with vertical bars representing the range. Figure S2. Average concentration of plasma cortisone following ZA administration (n = 8) with vertical bars representing the range. Figure S3. Average integrated peak area in plasma for 18‐hydroxycortisol following ZA administration (n = 8) with vertical bars representing the range. Figure S4. Average concentration of plasma 18‐HEPE following ZA administration (n = 8) with vertical bars representing the range. Figure S5. Average concentration of plasma OEA following TA administration (n = 8) with vertical bars representing the range. Figure S6. Average integrated peak area in plasma for AEA following TA administration (n = 8) with vertical bars representing the range. Figure S7. Average concentration ratio for plasma HC/C following TA administration (n = 8) with vertical bars representing the range. Figure S8. Average concentration of plasma 18‐HEPE following TA administration (n = 8) with vertical bars representing the range. [file DTA-17-1107-s001.docx]

**Kathy Tou^1^**, Adam Cawley^2^ Glenys Noble^3^, Jaymie Loy^3^, David Bishop^4^, John Keledjian^5^, Kireesan Sornalingam^5^, Stacey Richards^5^, Shanlin Fu^1^

1. Centre for Forensic Science, University of Technology Sydney, Sydney, NSW, Australia
2. Racing Analytical Services Ltd, Flemington, VIC, Australia
3. School of Veterinary Sciences, Charles Sturt University, Wagga Wagga, NSW, Australia
4. Hyphenated Mass Spectrometry Laboratory, University of Technology Sydney, Sydney, NSW, Australia
5. Australian Racing Forensic Laboratory, Racing NSW, Sydney, NSW, Australia

Corresponding author’s email: [Kathy.Tou@student.uts.edu.au](mailto:Kathy.Tou@student.uts.edu.au)

Leading author’s email: [Kathy.Tou@student.uts.edu.au](mailto:Kathy.Tou@student.uts.edu.au)

**S1. Zoledronic Acid (ZA) Administration Study**

**Figure S1: Average integrated peak area in plasma for AEA following ZA administration (n=8) with vertical bars representing the range.**

**Figure S2: Average concentration of plasma cortisone following ZA administration (n=8) with vertical bars representing the range.**

**Figure S3: Average integrated peak area in plasma for 18-hydroxycortisol following ZA administration (n=8) with vertical bars representing the range.**

**Figure S4: Average concentration of plasma 18-HEPE following ZA administration (n=8) with vertical bars representing the range.**

**S2. Tiludronic Acid (TA) Administration**

**Figure S5: Average concentration of plasma OEA following TA administration (n=8) with vertical bars representing the range.**

**Figure S6: Average integrated peak area in plasma for AEA following TA administration (n=8) with vertical bars representing the range.**

**Figure S7: Average concentration ratio for plasma HC/C following TA administration (n=8) with vertical bars representing the range.**

**Figure S8: Average concentration of plasma 18-HEPE following TA administration (n=8) with vertical bars representing the range.**
